# Stigma against patients with HIV/AIDS in the rapid expansion of antiretroviral treatment in large drug injection-driven HIV epidemics of Vietnam

**DOI:** 10.1186/s12954-019-0277-7

**Published:** 2019-01-17

**Authors:** Phung Quoc Tat Than, Bach Xuan Tran, Cuong Tat Nguyen, Nu Thi Truong, Thao Phuong Thi Thai, Carl A. Latkin, Cyrus S. H. Ho, Roger C. M. Ho

**Affiliations:** 1 Institute for Global Health Innovations, Duy Tan University, Da Nang, 550000 Vietnam; 2 Institute for Preventive Medicine and Public Health, Hanoi Medical University, Hanoi, Vietnam; 3 Bloomberg School of Public Health, Johns Hopkins University, Baltimore, MD USA; 4 Center of Excellence in Behavioral Medicine, Nguyen Tat Thanh University, Ho Chi Minh City, Vietnam; 5 Department of General Planning and Department of Cardiology, Friendship Hospital, Hanoi, Vietnam; 6 Department of Psychological Medicine, National University Hospital, Singapore, Singapore; 7 Department of Psychological Medicine, Yong Loo Lin School of Medicine, National University of Singapore, Singapore, Singapore

**Keywords:** Vietnam, Stigma, Discrimination, HIV/AIDS, Antiretroviral therapy

## Abstract

**Background:**

Despite existing efforts to provide antiretroviral treatment (ART) for all HIV-diagnosed people, stigma deprives them of the highest attainable health status and challenges the effectiveness of ART program in Vietnam. This study aimed to assess five dimensions of HIV-related stigma and explore its associated factors among ART patients in a multisite survey. Implications of this study support the development of HIV policies to improve patients’ access, utilization, and outcomes of ART program toward the 90-90-90 goal in Vietnam.

**Methods:**

A total of 1133 ART patients who were recruited by convenience sampling method from 8 ART clinics in Hanoi and Nam Dinh in a cross-sectional study from January to August 2013. Multivariate logistic regression was employed to identify factors associated with stigmatization.

**Results:**

The majority of participants reported experiencing stigmatization due to shame (36.9%), blame/judge (21.6%), and discrimination (23.4%). Further, 91.5% of participants disclosed their HIV status with others. The likelihood of experiencing stigmatization did not only associate with the patients’ socioeconomic status (e.g., age, occupation, education) and HIV status disclosure, but also their health problems. Those with anxiety or depression and perceived lower quality of life were more likely to experience stigma.

**Conclusions:**

To maximize the efficiency of the ART program, it is essential to develop interventions that reduce stigma involving individuals, families, and communities, and recognize and address complex health problems especially those patients showing depressive symptoms. Increasing quality of life of HIV-positive patients by providing vocational training, financial, family, and peer support will reduce the likelihood of experiencing stigma.

## Background

The World Health Organization created 90-90-90 treatment goal as a global commitment: 90% of people living with HIV (PLWH) know their HIV status, 90% of HIV-positive people receive antiretroviral treatment (ART), and 90% of PLWH on treatment have their viral loads suppressed [1]. Since then, ART has been an integral part of HIV/AIDS control efforts to reduce disease burden and improve life expectancy of PLWH [2–4]. However, HIV-related stigma, which pertains to any discriminatory, prejudicial attitudes, and beliefs toward PLWH [4–7], remains the major challenge of the effectiveness of ART programs [4, 5, 8]. PLWH are subjected to stigma for its associated behaviors such as multiple sexual partners, injecting drugs, and having same-sex partners [9]. They are not only blamed for risky behaviors, but also are segregated for causing harm to society. Previous research among PLWH found that HIV disclosure was also associated with impaired psychological and emotional health conditions [9–12]. Fear of disclosure when accessing to HIV testing, care, treatment services, and associating peer support group may delay treatment initiation and hinder treatment adherence [4, 5, 7, 12].

Vietnam has made a significant progress in HIV/AIDS epidemic response for the last 25 years: preventing newly infected cases, reducing HIV/AIDS-related mortality and fatality, and expanding harm-reduction interventions and HIV services. According to UNAIDS, in 2017, Vietnam has approximately 250,000 PLWH, 50% of those are receiving ART, and 43% of PLWH are virally suppressed [13]. With these statistics, Vietnam is far from meeting its commitment which is to expand HIV treatment by 2020, then become the first country in Asia to meet 90-90-90 goal, and end the AIDS epidemic by 2030. As Vietnam becomes a middle-income country, many international donations are shrunk and slowly withdrawn from the country. Vietnam is trying to include HIV services in national health insurance with the hope of reducing stigma and discrimination of PLWH. However, the effectiveness of this strategy is still unknown because of the strong HIV stigma that remains in the community.

Vietnam has unique culture and social values that make HIV-related stigma toward PLWH more complex and dynamic. It is necessary to understand the phenomenon of bonding between individual and community in Vietnamese culture; socio-cultural values heavily affect HIV stigma. In literature, stigma toward PLWH in Vietnam has been fueled by the misconception about HIV, societal rejection, and judgment on HIV-related risky behaviors based on cultural and religious norms [4–6, 12, 14–16]. Since the very first attempt to raise awareness of and control HIV/AIDS, HIV/AIDS was portrayed as social evils to scare people away from risky behaviors [17]. With the given information on posters and banners, people believed HIV/AIDS was a death sentence. People became afraid of HIV infection and avoided any types of contacts with HIV-infected patients. Recently, even though the Government of Vietnam has shifted from a punitive to harm-reduction approach, the taboo regarding HIV/AIDS remained. In addition, due to cultural and religious norms based on sexual orientation and sexual behaviors, the key HIV populations such as sex workers, drug user, and men who have sex with men are stigmatized even more because they are social evils, criminals, and immoral.

At the same time, despite many non-discrimination laws that have created to protect PLWH, these laws lack enforcement when incidents of violence [12], denial of care [6, 12, 16], or hate crimes happen [5]. As the Government of Vietnam shifted from punitive to harm-reduction approach, sex worker and drug users are no longer sent to jail. Instead, they are sent to detention camps and rehabilitation centers where they are still within their communities and family members can visit them. Even though they are not considered as criminals, the fact that they are going to detention camps and rehabilitation centers indirectly disclose their behaviors and causes rumors that they have HIV/AIDS. Besides, in healthcare sector, HIV test results of patients were not always kept confidential, especially in lower health care administration [12]. Moreover, in a family-oriented culture like Vietnam, HIV/AIDS becomes a family disease which means not only the individuals but also their family and relatives are blamed, judged, and discriminated [12]. Previous research also found that PLWH isolated themselves to avoid stigma or discrimination, and perceived greater loneliness [9]. For a long time, it creates a chronic level of stress which causes psychological (e.g., anxiety, depression) and other health problems [4, 9, 18]. Because of all the following consequences and stigma, PLWH avoid disclosing their status with others.

HIV stigma deprives PLWH of the highest attainable health status in many ways, and it stands in the way of enhanced HIV services. In order to dispel stigma, we need to understand where it lies in the community. This study, therefore, aimed to explore current stigma in Vietnam experienced by ART patients regarding perception of being blamed or judged, feared of infection, ability to disclose HIV status, feeling shame, and discriminated. We also measured the degree of association between factors and perceived stigma. The findings may suggest directions to develop more effective policies and strengthen efforts to reduce stigma, improve treatment outcome and quality of life of ART patients, and to achieve 90-90-90 treatment goal.

## Methods

### Study setting and subjects

From January to August 2013, a cross-sectional study was conducted in Hanoi and Nam Dinh where two epicenters are providing HIV/AIDS control and treatment services in northern Vietnam. We selected eight outpatient clinics which met the following inclusion criteria: (1) the clinic belongs to the public health system in Vietnam (including central, provincial, and district levels), (2) the clinic provides ART service, and (3) the clinic implements their ART programs following the official guidelines from the Vietnamese Ministry of Health [19]. A total of eight outpatient clinics—five from Hanoi and three from Nam Dinh—were selected, including Bach Mai Hospital representing central administration level, Nam Dinh provincial hospital, and Nam Dinh provincial AIDS Control Centre representing provincial administration level, and five district health centers from Hoang Mai, Long Bien, Dong Anh, Ha Dong, Xuan, and Truong District.

Eligibility criteria for recruitment included (1) 18 years old and older, (2) having a confirmed HIV-positive test result, (3) enrolling or receiving ART at one of the selected clinics, (4) having no major cognitive impairment, and (5) agreeing to participate and providing written consent. The participants were excluded from the study if they had major cognitive impairment causing inability to answer the questionnaires. People who suffered from serious illness during the recruitment process were also excluded. The cognitive impairment and serious illness were determined by physician’s diagnosis. A total of 1133 participants were recruited by convenience sampling method, and response rate was 80–90% across all sites. The participants decided not to participate in the study because of time conflict, discomfort, and poor physical health during the time of study.

Eligible patients were invited into a small counseling room for the interview. Using a structured questionnaire, the participants were interview via face-to-face for 20 min. The interviewers were master students of public health at Hanoi Medical University. The students had experience working in HIV research study and were not affiliated with the participating clinics.

### Measures and instruments

With the given context of HIV/AIDS in Vietnam, it is necessary to examine HIV-related stigma using a contextualized measure instrument. Therefore, we created a more contextualized questionnaire instead of using previous international stigma scale. The outcomes of this study were measured by five indicators of HIV/AIDS-related stigma according to USAID: (1) blame, judgment; (2) shame; (3) enacted stigma/discrimination; (4) disclosure; and (5) fear of casual transmission and refusal of contact [20]. Then we piloted the questionnaires among PLWH before interviewing participants.

During the interview, the participants were asked if they had experienced any of the above types of stigma within 30 days. The response options included (1) Yes, (2) No, and (3) No answer.

1. In general, have you recently been blamed or judged because of your health status?
2. Do you currently feel shame because of your health status?
3. Have you felt discriminated against or treated badly by others? In which circumstances (work place/all health facilities/family/community/others)?
4. Have you ever disclosed your health status with others? With whom did you share?
5. Has anyone expressed fear of contracting HIV from casual contacts with you?

We also collected participants’ socioeconomic characteristics and ART treatment-related information. In addition, the participants were also asked whether they had peer support and attended peer-to-peer meeting.

#### Socioeconomic characteristic

The participants were asked to report age, gender, education level, marital status, religion, living place, and employment status.

#### ART treatment-related information

The participants were asked to report the latest CD4 cell count, HIV stage, ART duration, their needs of ART, whether they currently received treatment, and health-related quality of life (HRQOL). HRQOL was measured by using EuroQOL-5 dimensions-5 levels (EQ-5D-5 L) instrument in the Vietnamese version which was employed from a well-validated tool called EuroQol [21]. This tool assessed five domains including mobility, self-care, usual activities, pain/discomfort, and anxiety/depression. Each domain had five response levels: no problems, slight problems, moderate problems, severe problems, and extreme problems [22]. The combination of responses gives 3125 health index [21]. Furthermore, we employed 100-point visual analogue scale (EQ-VAS) to measure the self-reported HRQOL, ranging from 0 which was referred to “the worst health condition that you can imagine” to 100 which was referred to “the best health condition that you can imagine” [23].

### Statistical analysis

STATA version 12 (Stata Corp. LP, College Station, United States of America) was employed to analyze data. Descriptive analysis such as *t* test and chi-square were used to explore demographic characteristics of respondents as well as HRQOL, ART status, and stigmatization. A *p* value < 0.05 was used to determine statistical significance. Multivariate logistic regression was employed to identify factors associated with stigmatization. This strategy used threshold with the log-likelihood ratio test to have predictors with *p* values of < 0.2 included.

## Results

Table 1 describes the socioeconomic characteristics of the respondents. Out of 1133 participants, more than half of participants were male (58.7%), and the mean age was 35.3 (SD = 6.9). The majority of the participants had secondary level education (36.9%). Then, 20.4% of participants were currently unemployed; meanwhile, 41.1% of participants reported self-employed, 24.9% were workers or farmers, and 7.1% were white collars. More than three-quarters of participants lived in urban area (77.2%), and practiced ancestral worship (88.4%).

**Table 1:** Socioeconomic characteristic of respondents

	Single		Live with partner		Total		*p* value
	*n*	%	*n*	%	*n*	%	
Gender							
Male	205	46.6	460	66.4	665	58.7	< 0.01
Female	235	53.4	233	33.6	468	41.3	
Education							
Illiterate	4	0.9	8	1.2	12	1.1	0.48
Elementary	90	20.5	130	18.8	220	19.4	
Secondary	171	38.9	247	35.6	418	36.9	
High school	135	30.7	227	32.8	362	32.0	
Vocational	15	3.4	39	5.6	54	4.8	
University	25	5.7	42	6.1	67	5.9	
Living place							
Rural	80	18.2	178	25.7	258	22.8	< 0.01
Urban	360	81.8	515	74.3	875	77.2	
Religion							
Cult of ancestors	382	86.8	619	89.3	1001	88.4	0.02
Buddhism	23	5.2	32	4.6	55	4.9	
Catholic	27	6.1	41	5.9	68	6.0	
Protestant	8	1.8	1	0.1	9	0.8	
Employment							
Unemployed	110	25.0	121	17.5	231	20.4	0.01
Self-employed	165	37.5	304	43.9	469	41.4	
White collars	23	5.2	57	8.2	80	7.1	
Workers, farmers	110	25.0	172	24.8	282	24.9	
Others	32	7.3	39	5.6	71	6.3	
	Mean	SD	Mean	SD	Mean	SD	
Age	34.7	0.3	36.0	0.3	35.5	6.9	0.50

Regarding health-related quality of life, the most commonly reported problem was anxiety or depression (44.9%), followed by pain or discomfort (37.7%), and mobility (20.5%). The average VAS score on quality of life was 68.8 (SD = 17.3). These health problems were reported more among single HIV patients than those living with a partner (Table 2).

**Table 2:** Self-reported health-related quality of life

	Single		Live with partner		Total		*p* value
	*n*	%	*n*	%	*n*	%	
Self-reported health problems							
Mobility	107	24.3	125	18.0	232	20.5	0.01
Self-care	48	10.9	62	9.0	110	9.7	0.28
Usual activities	85	19.3	103	14.9	188	16.6	0.05
Pain or discomfort	194	44.1	233	33.6	427	37.7	< 0.01
Anxiety or depression	237	53.9	272	39.3	509	44.9	< 0.01
	Mean	SD	Mean	SD	Mean	SD	
VAS	68.3	0.8	69.1	0.6	68.8	17.3	0.50

Table 3 illustrates that 42.1% of patients reported no HIV symptoms (42.1%), and 96.0% patients currently using ART. The average CD4 cell count was 295 (SD = 215), and the average ART duration was 3.5 years (SD = 2.2). A half of respondents participated in peer-to-peer meeting (50.6%); however, only 34.5% of participants perceived having support from their peers.

**Table 3:** ART status

	Single		Live with partner		Total		*p* value
	*n*	%	*n*	%	*n*	%	
HIV stage							
No symptom	185	43.4	271	41.3	456	42.1	0.15
Have symptom	86	20.2	107	16.3	193	17.8	
AIDS	33	7.8	68	10.4	101	9.3	
Unknown	122	28.6	210	32.0	332	30.7	
Need ART							
Yes	424	96.8	669	96.7	1093	96.7	0.91
No	14	3.2	23	3.3	37	3.3	
Using ART							
Yes	403	95.1	647	96.6	1050	96.0	0.21
No	21	5.0	23	3.4	44	4.0	
Peer-to-peer meeting							
No	198	46.9	346	51.0	544	49.5	0.19
Yes	224	53.1	332	49.0	556	50.6	
Have peer support							
Yes	166	37.7	225	32.5	391	34.5	0.07
No	274	62.3	468	67.5	742	65.5	
	Mean	SD	Mean	SD	Mean	SD	
CD4	281	212	303	217	295	215	0.49
ART duration (year)	3.5	2.3	3.4	2.2	3.5	2.2	

Table 4 shows different types of stigmatization experienced by participants. Further, 36.9% of participants reported experiencing shame, stigmatization blame/ judge (21.6%), and discrimination (23.4%). Majority of participants were able to disclose their HIV status with others. There were more ART patients living with a partner reported disclosure than who were single. Single participants reported higher rate of stigma events than those who lived with partner.

**Table 4:** Perceived stigmatization among ART patients

	Single		Live with partner		Total		*p* value
	*n*	%	*n*	%	*n*	%	
Stigmatization							
Blame/Judge	94	22.9	138	20.9	232	21.6	0.43
Shame	164	39.9	232	35.1	396	36.9	0.11
Discrimination	123	28.0	142	20.5	265	23.4	< 0.01
Disclosure	386	87.7	651	93.9	1037	91.5	< 0.01
Fear of HIV infection	129	29.4	198	28.6	327	28.9	0.78

Regression models in Table 5 indicate that having white collars jobs (OR = 0.17, Cl = 0.005–0.58), not knowing HIV status (OR = 0.49, Cl = 0.33–0.74), quality of life were negatively associated with perceived discrimination. Meanwhile, suffering from pain or discomfort (OR = 1.73, Cl = 1.17–2.56) was positively associated with perceived discrimination. Individuals with anxiety and depression were more likely to feel shame (OR = 1.77, Cl = 1.29–2.41), discriminated (OR = 1.74, Cl = 1.18–2.58), and being mistreated due to the fear of HIV infection (OR = 1.42, Cl = 1.03–1.97). Having unstable jobs (OR = 2.33, Cl = 1.30–4.18) increased the likelihood of perceived shame, while being younger (OR = 0.96, Cl = 0.94–0.99) increased the likelihood of perceived blame or judgment. Having mobility problems (OR = 1.88, Cl = 1.20–2.93) did not only increase the likelihood of HIV patients being blamed or judged, but also decreased the likelihood of HIV status disclosure (OR = 0.49, Cl = 0.27–0.91).

**Table 5:** Associated factors

	Blame/Judge		Shame		Discrimination		Disclosure		Fear of HIV infection	
	OR	95 CI	OR	95 CI	OR	95 CI	OR	95 CI	OR	95 CI
Gender (Male–ref)										
Female					1.36*	(0.95–1.93)				
Education (Illiterate)										
Secondary school							0.37***	(0.21–0.68)		
High school			0.35**	(0.14–0.86)			0.22***	(0.07–0.70)		
Marital status (Single–ref)										
Live with spouse/partner					0.72*	(0.51–1.01)	1.91**	(1.10–3.33)		
Religion (Cult of ancestors–ref)										
Buddhism									2.60***	(1.32–5.12)
Catholic			1.77*	(0.97–3.23)			0.35**	(0.15–0.80)		
Employment (Unemployed–ref)										
White collars					0.17***	(0.05–0.58)				
Workers, Farmers			1.34*	(0.95–1.88)	1.39*	(0.95–2.03)				
Other			2.33***	(1.30–4.18)					0.51*	(0.24–1.06)
Self-reported health problems										
Having problem with mobility (Yes vs No)	1.88***	(1.20–2.93)					0.49**	(0.27–0.91)		
Pain or discomfort (Yes vs No)					1.73***	(1.17–2.56)				
Anxiety or depression (Yes vs No)			1.77***	(1.29–2.41)	1.74***	(1.18–2.58)	0.23***	(0.11–0.48)	1.42**	(1.03–1.97)
HIV period (No symptom–ref)										
Have symptom			1.47*	(0.99–2.18)						
AIDS					1.70**	(1.02–2.85)				
Unknown			0.72*	(0.51–1.00)	0.49***	(0.33–0.74)	2.21**	(1.06–4.58)	0.54***	(0.38–0.77)
Peer-to-peer meeting (No–ref)										
Yes									0.73**	(0.54–0.98)
Disclosure (No–ref)										
Yes					3.82***	(1.84–7.95)			3.21***	(1.59–6.50)
Age	0.96***	(0.94–0.99)	0.98*	(0.96–1.00)						
VAS	0.97***	(0.96–0.98)	0.99***	(0.98–0.99)	0.98***	(0.97–0.99)			0.98***	(0.97–0.99)

***p < 0.01; **p < 0.05; *p < 0.1

Regarding HIV status disclosure, HIV patients who had high school education level (OR = 0.22, Cl = 0.07–0.70) and reported having anxiety or depression (OR = 0.23, Cl = 0.11–0.48) were less likely to disclose their HIV status. In term of religion, PLWH who were Catholics would more likely to conceal their HIV status (OR = 0.35, Cl = 0.15–0.80). At the same time, HIV patients those lived with a spouse or partner would more likely to disclose their HIV status (OR = 1.91, Cl = 1.10–3.33).

HIV patients who disclosed their HIV status were more likely to perceive discrimination (OR = 3.82, Cl = 1.84–7.95) and fear of HIV infection (OR = 3.21, Cl = 1.59–6.50). Moreover, those practiced Buddhism (OR = 2.60, Cl = 1.32–5.12) also reported higher odds of perceiving avoidance due to the fear of HIV infection. In contrast, those who did not know their HIV stage (OR = 0.54, Cl = 0.38–0.77) and attended peer-to-peer meeting (OR = 0.73, Cl = 0.54–0.98) were less likely to experience stigmatization related to the fear to HIV infection.

## Discussion

Our study enriches the existing understanding of stigmatization experienced by ART patients in Vietnam. Considering it in a context of a family-oriented and closely bonded community, our study found that the likelihood of experiencing stigmatization was not only associated with the patients’ socioeconomic and HIV status but also their health problems. Socioeconomic characteristics were associated with perceived shame, discrimination, ability to disclose HIV status, and fear of HIV infection. The results also indicated that patients who disclosed HIV status were more likely to perceive discrimination and fear of HIV infection. Moreover, those with lower quality of life were at higher risk of perceived stigmatization across all five measured dimensions. Notably, our results could be used as effective approaches to dispel stigma barrier and improve quality of life of PLWH in Vietnam.

### Stigma

Majority of participants reported perceived shame and fear of infection which is similar to what Gaudine et al. found in a study in 2010 [17]. In Vietnam, individuals with HIV were avoided, perceived anger and social rejection, and were viewed as a social ill. Their family members were shunned by neighbors, viewed as bad parents, experience financial hardship, and fear of HIV transmission. Community members and health professionals believed that HIV ruined family reputation, and they also avoid contact with HIV individuals [17]. HIV-related stigma causes PLWH faced problems of getting a job, receiving unfair treatment at work, and experiencing discrimination in healthcare setting [24]. After 8 years with extensive efforts, the experience associated with HIV-stigma remained till today.

### Associated factors

#### Socioeconomic characteristics

Our findings showed that patients who were younger were more likely to perceive blame or judge. Compared to older population, younger individuals work and involve in more social activities. Thus, they interact with others and are more prone to stigma. In Vietnamese culture, younger population, especially youth, is supposed to be pure. Because of HIV positive serostatus which could be due to mother-to-child transmission, the younger PLWH are often blamed or socially excluded [12, 25]. People believe that their family members or they commit a sin, so they are punished.

Similarly, having unstable jobs increased the likelihood of experiencing shame [26]. In Vietnamese culture, people often feel shame for not providing financial support to their family. Especially in the culture where everyone in the community connects, having unstable jobs also shames PLWH in the eyes of community [5] because the neighbors or others will questions why the individuals often quit their jobs. Unstable jobs also result from lower education level; PLWH suffer from stigma at school and quit early. Previous studies also found that PLWH have difficulty finding a job for long term partially due to HIV-related stigma at work from employers and co-workers [17]. It could be also caused by the time conflict between ART clinic and their work hours. On the same side, we found that white collars were less likely to perceive discrimination, which is consistent with previous study [4]. This could be explained by the fact that white collars usually work with highly educated co-workers and their workplace complies with HIV-privacy laws.

In the present study, the likelihood of HIV status disclosure was negatively correlated with education level. Unlikely, the previous research found those who were educated more would be more likely to reveal HIV status [27]. This could be explained by the fact that those with higher education are well educated about HIV, control their conditions, and cope with HIV better [28]. Therefore, they may not feel necessary to disclose their HIV status to others.

#### Health problems

Our study found that quality of life was negatively associated with stigma. Quality of life does not only pertain to living or health conditions but also perception. In previous studies, quality of life of PLWH was extensively examined and found to be a risk factor of perceived and enacted stigma, physical and mental health problems [8, 9, 12, 29]. Numerous studies have found the bidirectional relationship between anxiety or depression and HIV-related stigma [9, 18]. In our study, having anxiety and depression decreased the likelihood of disclosing HIV status and increased the likelihood of perceiving shame, discrimination, and fear of HIV infection. Previous research found that the risk of depression was four times higher among those who did not disclose their HIV status than their counterpart [18]. In a culture like Vietnam where people live closely in a community, HIV-related stigma makes PLWH constantly worried and anxious because they do not know how they will be treated and what people think about them. Especially after seeing how other HIV-infected individuals are treated, those have yet disclosed would kept confidential to avoid stigma. At the same time, PLWH are often isolated by community, or PLWH isolate themselves to avoid being stigmatized and discriminated [9]. Thus, the individuals develop negative feelings or emotions [18] and avoid anything associated with HIV/AIDS such as accessing to testing and HIV treatments [7]. Notably, this particular population deserves more attentions from community and clinicians.

In addition, our findings also showed that patients who had mobility problems would experience more blame or judgment. Meanwhile, perceived discrimination was reported more among those with pain or discomfort and AIDS [29]. Moreover, Vietnamese people believe in “You reap what you sow.” People believe that having physical problems, pains, or disgusting AIDS symptoms are consequences of PLWH’s immoral behaviors. Also, either mobility problems, pain and discomfort, or AIDS symptoms exhibit outside; thus, others could easily see, and then judge or discriminate the individuals.

#### HIV status disclosure

Disclosing HIV status potentially helps to control HIV/AIDS epidemics by preventing HIV infection, increasing access to HIV-related services, and ensuring treatment adherence [30]. However, the decision to disclose HIV status is challenging to PLWH due to stigma and the fear of HIV infection [31, 32]. In this study, those did not know their HIV status and live with spouse or partners were more likely to disclose their HIV status. This could be explained by the fact that PLWH choose to disclose their HIV status to receive testing, treatments, and prevent HIV infection [3, 29, 30]. Previous study found that disclosure increases the likelihood of safe sex to keep the partner from infection [33, 34]. However, some people choose to disclose their HIV status to engage in unprotected sex with other HIV-positive partners [35].

Our results also indicated that those who disclosed HIV status had almost four times more likely to report perceive discrimination and fear of infection than those did not disclose, which is consistent with previous research [5, 31, 35, 36]. Discrimination might be the rejection from sexual partner, family, friends, and community because they are afraid of infection [35]. Eventually, it will affect treatment adherence and health outcomes [6, 12, 32, 37].

#### Peer support

In this study, peer-to-peer meeting had no association with either perceived blame/judge, shame, discrimination, or HIV disclosure. However, peer-to-peer meeting decreased the odds of experiencing fear of infection from others. Being in a group of people who share a common experience or diagnosis provides HIV-positive individuals sense of social acceptance, reciprocal support, and empowerment [38]. As their self-esteem and self-confidence arise, they perceive less fears, uncertainties, and stigma [39].

#### Implications

We found that individuals with unstable jobs were three times more likely to perceive shame. Providing convocational training and appropriate jobs to stabilize income of PLWH will benefit them and their family. Instead of perceiving shame when they are financial burden of family, having a job gives individuals a sense of pride and success. Being employed and having a secured job significantly improved physical and mental health-related quality of life among adults living with HIV [40]. In addition, anti-HIV-related discrimination regulations should be enforced at workplaces. Posters and formal announcements should be made to employer and employees. This will allow PLWH to reintegrate into community as they work, and encourages them to access HIV-related care services when they less worry about what to do for living.

Providing peer support reduces the stigma associated with HIV and empower HIV-positive individuals [38, 39]. However, our findings indicated that our current peer support program was not effective enough to reduce HIV-related stigmatization in Vietnam. We might need a more holistic approach to create a social support system within community and society: increase knowledge and awareness, create positive attitudes, and implement social support. Mass media campaigns sustainably increase health-related knowledge and produce positive changes across large populations [41]. Previous studies demonstrated the positive correlation between mass media campaigns and condom use, HIV transmission, and prevention knowledge [42, 43]. In Vietnam, HIV-related stigma is largely due to the misconception about HIV. Thus, the information campaigns will help to correct the wrong information and promote positive attitudes toward HIV/AIDS and PLWHA. However, mass campaigns can only have an effect on certain segments of population due to knowledge gap between those with high and low education level [44]. Therefore, it is important to ensure the mass media campaign is available and accessible, and the presenting information is tailored to different segment of population. Also, in illiterate or low health literacy community, community-based participatory programs and interpersonal community activities using social and religious networks can be useful [45]. This comes from the compassion and sympathy of society, family, and beloved ones toward ART patients.

An ultimate goal of reducing stigma among PLWHA is to improve their health. However, more efforts are required to improve health status of individuals with chronic illness, especially with stigmatized illness and depressive symptoms. First, physicians should screen for depressive symptoms among PLHWA, and provide counseling. Second, we should develop community-based interventions to promote psychosocial well-being among PLWHA. Wu and Li found that support group which provides emotion and information support, mentoring, and community involvement increased positive social interaction, and reduced HIV/AIDS-related stigma, distress, depression, anger, and anxiety [46, 47]. These community-based interventions should consider contextual factors, have high frequency of follow ups, and last for a long period of time [48]. As individuals improve their mental health and perceive social acceptance, they will be in charge of their health, stay adhered to their treatment, and improve health outcomes.

### Strengths and limitations

The study recruited large amount of participants from Hanoi and Nam Dinh which are epicenters of HIV epidemics in Vietnam. However, there are several limitations that need to be considered when interpreting the results. First, health problems and stigmatization were self-reported. The information could have been under- or over-reported due to recall and social desirability response bias. Second, due to the nature of cross-sectional study, the study could not establish the causal relationship between stigmatization and its associated factors. This problem could be eliminated if researcher could perform longitudinal study. Third, the participants were recruited by convenience sampling method which may reduce the generalizability of the study.

## Conclusion

In conclusion, our findings illustrated that stigma was not only associated with socioeconomic characteristics but also other health problems. HIV status disclosure also increased the likelihood of discrimination and stigmatization related to fear of HIV infection. There is a need of interventions targeting PLWH with anxiety and depression. Increasing quality of life of PLWH by providing vocational training, financial, and family and peer support will decrease the likelihood of experiencing stigmatization.

## Abbreviations

AIDS: Acquired immune deficiency syndrome
ART: Antiretroviral treatment
EQ-5D-5 L: EuroQOL-5 dimensions-5 levels
EQ-VAS: Visual analogue scale
HIV: Human immunodeficiency virus
HRQOL: Health-related quality of life
PLWH: People living with HIV
